# Interstitial cells of Cajal, from structure to function

**DOI:** 10.3389/fnins.2013.00043

**Published:** 2013-04-01

**Authors:** Jan D. Huizinga, Ji-Hong Chen, Hanne B. Mikkelsen, Xuan-Yu Wang, Sean P. Parsons, Yong Fang Zhu

**Affiliations:** ^1^Department of Medicine, Farncombe Family Intestinal Health Research Institute, McMaster UniversityHamilton, ON, Canada; ^2^Department of Gastroenterology and Hepatology, Wuhan University Institute of Digestive and Liver diseases, Renmin Hospital of Wuhan UniversityWuhan, China; ^3^Department of Cellular and Molecular Medicine, University of CopenhagenCopenhagen, Denmark

**Introduction to**

**The ultrastructure of the muscle coat of the human gastro-oesophageal junction, with special reference to “interstitial cells of Cajal”**

a historical paper originally written in Italian and now translated by Faussone Pellegrini et al. (2013).

Ramon y Cajal, searching for a neural network simpler than the brain, studied the rabbit small intestine to find interstitial cells he considered the end cells of the sympathetic nervous system (Ramon y Cajal, 1911). His drawings of what would later be called interstitial cells of Cajal, are still captivating admiration for their accuracy. The hypothesis of ICC as nerve cells was based on the observation that ICC associated with the myenteric plexus of the rabbit intestine, stained with methylene blue and silver impregnation according to the Golgi method, similar to neural tissues. Furthermore, the cells appeared to intercalate between typical nerve cells and smooth muscle cells. In the years following the publications of Cajal, morphologists studied the ICC in the gut almost continuously coming to the conclusion that they were nerve cells, Schwann cells, fibroblasts or myoid cells. Occasionally someone connected the cells with the generation of the rhythmicity of gut motor activity. Keith saw structural similarities with sinoatrial node cells and hypothesized them to be pacemaker cells (Keith, 1915). Leeuwe wrote in 1937: “Interstitial cells of Cajal are the end formations of the sympathetic nervous system, responsible for the rhythmic contractions of the intestinal peristaltic activity” (Leeuwe, 1937). Ambache, although still believing that ICC belonged to the nervous system, nevertheless suggested that an electrical slow wave, preceding contractions “represent the discharge of a pacemaker in the gut, and may arise in the nerve net which was described by Cajal” (Ambache, 1947). Nelemans and Nauta commented: “Since most organs containing interstitial cells [of Cajal] show rhythmicity …. its seems to us most probable that we have to find the origin of this rhythmicity in the interstitial network” (Nelemans and Nauta, 1951). Electron microscopy heralded the modern era of research into the physiology and pathophysiology of interstitial cells of Cajal. The first paper that provided the hypothesis that ICC were pacemaker cells based on this new technique was published in an Italian journal by Professor Faussone-Pellegrini at the University of Florence, Italy, where she described ICC observed in esophageal and gastric specimen from patients not suffering from motility pathologies (Faussone Pellegrini et al., 1977). A translation of this paper in English has now appeared in Frontiers in Autonomic Neuroscience (Faussone Pellegrini et al., 2013). Faussone-Pellegrini graduated from the University of Florence in 1963 at the age of 23 and was offered a position in the Histology and Embryology Department to instruct the members of the department in the use of the new transmission electron microscope. With little money and no assistance Faussone Pellegrini discovered then (1967–1968) the ICC in the rat stomach, but could not publish anything because her professor decided that she was too young and had not been asked to look for something outside of the topic she should study. Years later, in the period of 1974–1976 Faussone-Pellegrini was asked by the surgeon Camillo Cortesini to look at specimen from the gastro-esophageal junction from patients with achalasia where she saw that the ICC morphology differed from controls as they had fewer organelles such as mitochondria, smooth endoplasmic reticulum and they had also fewer contacts with smooth muscle cells and nerves (Faussone Pellegrini et al., 1977). The fact that achalasia is associated with poor peristalsis gave her ideas for the hypothesis that ICC might be pacemaker cells. The development of the hypothesis was also helped by correlating physiological findings from the literature with the structural information she was discovering. Faussone-Pellegrini (Faussone Pellegrini et al., 1977) refers to two chapters from the 1986 Handbook of Physiology. Holman wrote that pacemaker activity was likely generated by a few or all longitudinal muscle cells in the small intestine, acknowledging that it was unlikely that all smooth muscle cells exhibited pacemaker properties (Holman, 1968). Prosser and Bortoff also focused their attention on longitudinal muscle cells but they do make the following statement: “On morphological grounds, Tiegs (1925) postulated that the interstitial cells which Cajal had described as abundant along nerves … form an interstitial net that originates, conducts, and coordinates rhythmic contractions.” However, Prosser and Bortoff appeared to dismiss this by the statement that “Richardson (1958) clearly showed by electron microscopy that they are fibroblasts forming sheaths around nerves.”

In addition to physiological studies, comparative morphology helped Faussone-Pellegrini to create the hypothesis. Faussone-Pellegrini writes: “The low degree of differentiation of interstitial cells as contractile elements might be linked to self-excitation, as in myocardium (Viragh and Challice, 1973), where the specific tissue devoted to generation and conduction of impulses is made up of cells that are less well differentiated for contraction than common myocardiocytes” (Faussone Pellegrini et al., 2013).

Since the original paper was written in Italian (Faussone Pellegrini et al., 1977) it did not receive a wide audience and Faussone-Pellegrini was anxious to publish in English. The objective was a study of ICC in the human small intestine. The first attempts to publish were unsuccessful as reviewers believed the ICC to be immature muscle cells or poorly fixed muscle cells. But finally in 1983 her study was published where she described ICC in the myenteric plexus area and in the deep muscular plexus area (Faussone Pellegrini and Cortesini, 1983). During this time Faussone Pellegrini also studied pre- and post-natal mouse intestine and demonstrated that the ICC were not immature smooth muscle cells and provided information on the morphology of differentiating ICC from the mesenchymal cell to “adult” ICC (Faussone Pellegrini, 1984).

The work of Faussone-Pellegrini became better known through the publications and conference presentations of Professor Lars Thuneberg at the University of Copenhagen, Denmark. Faussone-Pellegrini's (1977) paper was quoted in the seminal doctoral thesis of Thuneberg (1982), culminating years of electron microscopy on an old Hitachi microscope. Thuneberg had the ability to observe details that appeared irrelevant to most observers, which provided him with a wealth of ideas and many original hypotheses. Thuneberg discovered the ICC around 1974 but free of the pressure to publish, it was not until 1982 that the research appeared as a doctoral thesis (Thuneberg, 1982). Thuneberg expanded on the structural evidence for ICC as pacemaker cells and he soon provided the first physiological evidence together with Juri Rumessen (Thuneberg et al., 1984). Slow wave activity had been shown to be derived from the myenteric plexus area, so it was decided to investigate the possibility that a photochemical ablation of the ICC-MP network would cause the disappearance of recordable slow wave activity. Vital methylene blue happened to be uniquely accumulating in the ICC-MP network and when the cells were exposed to direct illumination, the ICC were severely injured and it did cause disappearance of the slow waves (Thuneberg et al., 1984), providing strong support for the idea that the ICC-MP are intestinal pacemaker cells. The physiologist who had come closest to predicting this outcome was Tomita who published in 1981: “It is thus possible that some particular cells located between the muscle layers act as pacemakers for the slow waves, and activate both the longitudinal and circular muscles” (Tomita, 1981).

The study of the photochemical ablation was communicated at the 9th International GI Motility Meeting in Aix en Provence (Thuneberg et al., 1984) which stimulated several laboratories to start working on ICC. Thereafter the number of publications on ICC rose dramatically (Thuneberg, 1999). Although Szurszewski does not mention ICC as a possible source in the 1981 “bible” of gastrointestinal physiology: “The physiology of the Gastrointestinal Tract” edited by Leonard Johnson (Szurszewski, 1981), in 1986, Szurszewski's laboratory records electrical activity of isolated sections of the small intestine and concludes that spontaneous slow waves of the small intestine of the dog, cat, rabbit, opossum, and human are generated in non-neural cells located between the longitudinal and outer circular muscle layer. It is suggested that ICC might be the source (Hara et al., 1986). Suzuki et al. did similar studies in the cat jejunum and came to the same conclusion (Suzuki et al., 1986). In the 1987 edition of “Physiology of the gastrointestinal tract,” Thuneberg's 1982 paper is extensively discussed and Szurszewski writes: “As to the nature of these cells [generating slow waves] … the interstitial cells of Cajal seem to hold the greatest amount of promise (Szurszewski, 1987). The ICC had been accepted into the bastion of gastrointestinal physiology! In 1989 more physiological evidence from other laboratories confirmed the pacemaker role of ICC (Barajas-Lopez et al., 1989; Langton et al., 1989). In 1999 Thuneberg and Faussone Pellegrini published a joint paper, the “Guide to the identification of interstitial cells of Cajal” (Faussone-Pellegrini and Thuneberg, 1999).

**Figure 1:**
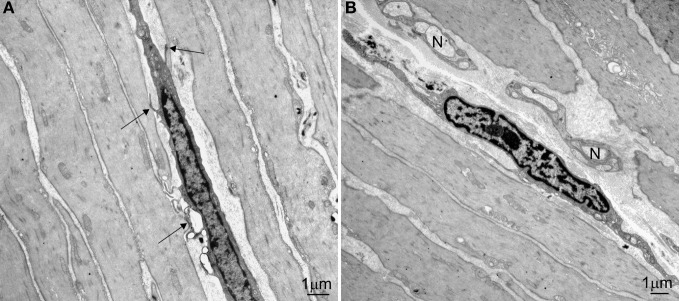
**Ultrastructure of ICC-IM in the circular muscle layer of the human lower esophagus. (A)** An ICC-IM and its processes form multiple connections (arrows) with adjacent smooth muscle cells. **(B)** An ICC-IM in a small septum is close to two small nerve bundles (N).

It was peculiar that Faussone Pellegrini developed the idea that ICC were pacemaker cells governing peristalsis while working on the *esophagus*, where under normal conditions no spontaneous rhythmic activity is noted and where peristalsis is assumed to be under vagal control. Indeed, all subsequent physiological studies on the role of ICC as pacemaker cells did not involve the esophagus. Loss of peristalsis of the esophageal body is thought to be due to loss of neurons (Kraichely and Farrugia, 2006) or due to LES dysfunction (Kraichely and Farrugia, 2006). The esophagus has very few ICC associated with the myenteric plexus, the ICC most often associated with pacemaker activity. The esophagus has abundant intramuscular ICC (ICC-IM) dispersed throughout the circular and longitudinal muscle (Figure 1). ICC-IM are thought to be involved in pacemaking and slow wave propagation in the stomach (Hirst et al., 2006). Interestingly, ICC are also found in the striated muscle of the esophagus (Faussone-Pellegrini and Cortesini, 1986). Are esophageal ICC associated with peristalsis? As elsewhere in the body, the esophagus has overlapping mechanisms of propulsion. Swallow induced propulsion is directed and coordinated by sequential excitation through vagal fibers programmed by the swallowing center in the central nervous system. In the absence of vagal activity, the intramural neural mechanism can take over. Swallowing a bolus activates this system and the subsequent propagating contraction has very similar characteristics as the one directed by the central nervous system (Diamant, 1989). Direct stimulation of the esophageal muscle in the opossum produces contractions that propagate in a peristaltic manner at a velocity that is similar to that of peristaltic contractions produce by swallows, in the presence of TTX (Sarna et al., 1977). Hence the esophagus has a myogenic control system that can fully orchestrate peristaltic activity and the network of ICC is a logical candidate for its origin. In some patients with achalasia, strong rhythmic contractile activity is noted clearly indicating the presence of a pacemaker (Jee et al., 2009). New evidence in the human esophagus suggests that the pacemaker might be a network of ICC-IM and PDGFRα positive cells (Ji-Hong Chen and Jan D. Huizinga, unpublished). Hence, Faussonne Pellegrini's idea of ICC in the esophagus as pacemakers might still be proven correct.

In summary, the hypothesis that ICC are pacemaker cells of the gut has appeared in the literature since 1915. Faussone-Pellegrini was the first to publish a study in 1977 that strengthened the hypothesis through the use of electron-microscopy. This notion was further developed and popularized by Thuneberg in 1982, and this started the modern era of physiological studies into the cellular origins of gut pacemaker activity.
